# 

**DOI:** 10.1192/bjb.2019.21

**Published:** 2019-08

**Authors:** Sabina Dosani

**Affiliations:** Consultant Child and Adolescent Psychiatrist, Department of English, Virginia Woolf Building, Kings College London, 22 Kingsway, London WC2B 6NR, UK. Email: sabina.dosani@kcl.ac.uk

Lynne Jones has spent much of the last two decades setting up and running mental health programmes in places devastated by natural disasters and wars. Jones is a child psychiatrist, who began her career in the Allinton Psychiatric Hospital, the former Allinton Asylum. From the off, she cared more about pragmatic pacifism and ‘doing the right thing’, which included a commitment to living by her values of public service and altruism. She has a CV ‘full of gaps’: when she was at Greenham Common, Bosnia, on the Somali border in Ethiopia, in Kosovo, Ethiopia, Haiti, Tacloban, and many other places where bombs have fallen or tsunami have washed away communities. Jones says her mother thinks she is on ‘an extended gap year’.

Her memoir has been compared to the writing of Oliver Sacks, which I think does them both a disservice. Jones' style and approach are different to Sack's pen portraits of neurological conundrum, although she does interlace clinical stories through her accounts. Her reminiscences of being with asylum patients on the dance floor evoke the recent work of Dr Charlie Howard, whose Music and Change project engages with teenage gangs at street level, co-producing models of care delivery.

Professionally, there is much to admire here, not least Jones' commitment to academic rigor despite being far from university departments. For instance, she describes staying on in Bosnia for an additional year after her contract had ended to study the effects of war on children. Her contributions to the intellectual, cultural and academic life in the countries she is sent to are similarly admirably. These exchanges are two-way: by spending time with communities and by listening to young people, Jones provides solutions that work for them and their families. She wears diverse theoretical cloaks lightly, using combinations of systemic family therapy, medication, group therapy and supportive counselling. Her stance is pragmatic, for example commencing a young man on medication earlier than she would like so that she can monitor the effects before her likely evacuation from the country.

Jones writes without bitterness or naïvety, recognising that, as a humanitarian worker, she is sometimes a pawn in political game, for example when she is part of a wider evacuation of international aid workers and has to say hurried farewells to unwell patients and local colleagues. There must surely have been times when Jones was fatigued, professionally isolated, personally lonely. We don't hear about it. But though she is stoic, she is also angry, especially about the pseudoscience that underpins the trauma industry and the resultant thoughtless ‘quick-fixes’ from many who ought to know better.

The atrocities she describes are apocalyptic in both scale and terror. In them, she endures personal hardships: living without running water, without electricity, yet aware of her privileged status, her passport, her choice to be there, her safe home in the UK. Jones is repeatedly drawn back to disaster scenes, not because of their horrors, but because, ‘I hope,’ she says, ‘that some [of their courage] will rub off on me’. In retelling these stories from her long humanitarian career, she passes some of that courage to her readers. Her beautifully weft stories of a lone psychiatrist bearing children's unbearable burdens are beacons of hope to their bomb-shattered childhoods and to our broken world.

